# Environmental and occupational respiratory diseases – 1040. Associations between asthma and bronchial hyper-responsivness with allergy and atopy phenotypes in urban black South African teenagers

**DOI:** 10.1186/1939-4551-6-S1-P39

**Published:** 2013-04-23

**Authors:** Michael Levin, Rudzani Muloiwa, Cassim Motala

**Affiliations:** 1 Red Cross Hospital, University of Cape Town, South Africa

## Background

Epidemiological studies in South Africa show increasing prevalence rates of asthma and allergic sensitisation in both urban and rural Black African communities, and narrowing of the urban-rural gradient.

There is a paucity of current data on bronchial hyper-responsiveness (BHR) in urban Black African children, associations between asthma and BHR and the relationship between BHR, allergen sensitisation and other atopic diseases.

## Objectives

To determine asthma and allergy phenotypes in unselected urban Black African (Xhosa-speaking) teenagers and to associate BHR with asthma, other atopic diseases and allergen sensitisation.

## Methods

Cross sectional study of two hundred and eleven urban high school Xhosa children. Modified ISAAC questionnaires regarding asthma, eczema and rhinitis were administered. BHR was assessed by methacholine challenge using hand-held nebulisers. Skinprick tests (SPT) were performed to 8 aeroallergens and 4 food allergens.

## Results

Asthma was reported in 9% and 16 % demonstrated BHR. Rhinitis was reported in 48% and eczema in 19%. Asthma was strongly associated with BHR. Asthma was associated with eczema whereas BHR was associated with rhinitis.

SPTs were positive in 34% of subjects; aeroallergens in 32%, food allergens 5%. The most common sensitivities were to house dust mites (HDM) and German cockroach. BHR was associated with sensitivity to any aeroallergen, Cat, HDM, Cockroach and Bermuda grass. Number of positive SPTs was associated with asthma and BHR. With each level of SPT positivity there is 40% increased prevalence of asthma and 70% increased prevalence of BHR.

The rate of allergen sensitisation in subjects with BHR (72%) was much higher than those without BHR (28%); house dust mite sensitivity: 69% in subjects with BHR and 18% in those without.

## Conclusions

These are the highest rates of allergen sensitisation in subjects with BHR documented in the African setting and the widest difference in sensitisation rates between subjects with and without BHR.

